# A One-Pot Three-Component Synthesis and Investigation of the In Vitro Mechanistic Anticancer Activity of Highly Functionalized Spirooxindole-Pyrrolidine Heterocyclic Hybrids

**DOI:** 10.3390/molecules25235581

**Published:** 2020-11-27

**Authors:** Raju Suresh Kumar, Dhaifallah M. Al-thamili, Abdulrahman I. Almansour, Natarajan Arumugam, Faruq Mohammad

**Affiliations:** Department of Chemistry, College of Science, King Saud University, P.O. Box 2455, Riyadh 11451, Saudi Arabia; 442106623@ksu.edu.sa (D.M.A.-t.); almansor@ksu.edu.sa (A.I.A.); anatarajan@ksu.edu.sa (N.A.); fmohammad@ksu.edu.sa (F.M.)

**Keywords:** multicomponent reaction, functionalized spirooxindole-pyrrolidine hybrids, anticancer activity, controlled cell death, apoptosis, ROS generation, caspase-3 activity

## Abstract

With an aim to develop more effective and affordable anticancer agents possessing a unique mechanism of action, we designed and synthesized derivatives of spirooxindole-pyrrolidine heterocyclic hybrids in good yields through a one-pot three-component (3+2) cycloaddition strategy. The synthesized compounds were characterized thoroughly for the physicochemical properties by making use of FT-IR, NMR spectroscopy, and mass spectrometry. Further, these compounds have been evaluated for the influence of anticancer activity against HepG2 cells up to 200 µg/mL concentration. The highly active molecular scaffold was tested for the in-depth mechanistic studies, and it was found that the major pathway of cell death is apoptosis which occurs through the induction of reactive oxygen species followed by the involvement of caspases.

## 1. Introduction

Carcinogenesis, also termed as tumorigenesis or oncogenesis, is a complex biological multi-step encompassing process involving a series of physiological mechanisms, where the outcome is the growth of cancerous tumors that has the capacity to completely arrest normal functions of an organ [1]. The inhibition of normal organ functions in cancerous cells, in general, first occurs through the cell division, followed by an uncontrolled growth of mutated cells to form malignant tumors, which on later stages spread to nearby cells and even migrate to different sites of the body by making use of the lymphatic system or bloodstream. To reduce the uncontrolled growth of malignant and fast dividing cells, many different antiproliferative drugs with enhanced pharmacological activity and bioavailability are used; however, the prolonged use of such drugs may develop uterine and endometrial cancers [2]. To overcome these issues, various synthetic and natural anticancer drugs are being developed and introduced into the market. As the nonselective action of such developed anticancer drugs are associated with many side effects including the bone marrow suppression, alopecia, and nephrotoxicity, the necessity of developing novel drug entrants with improved effectiveness to target only cancer cells is emphasized.

The construction of novel drug entrants with improved physiochemical properties, pharmacologic effectiveness, and no or less toxicity in cancer treatment has been an essential subject in the drug development sector. Undeniably, the construction of such molecular frameworks through a facile synthetic methodology with cost-effective considerations is still a challenge for medicinal chemists and necessitates long-lasting efforts. One of the developed strategies in recent years is the amalgamation of two or more diverse pharmacophores into a sole molecular framework, documented as molecular hybridization [3,4,5,6,7,8,9,10]. The molecular hybridization becomes an effective approach to the design of new drug entrants and is based on the identification of pharmacophoric structural subunits via the suitable combination of these subunits, which provides a base for the design of novel heterocyclic hybrids that preserves the preselected features of parental prototypes. The prominent advantage of employing the molecular hybridization approach is to trigger diverse targets by a sole molecule, thus increasing the therapeutic effectiveness and bioavailability profiles. Promisingly, noteworthy results with a diverse group of heterocyclic hybrids have been elegantly recognized as a result of this approach [11,12,13,14,15,16,17,18].

Spiropyrrolidine oxindoles hold fascinating structural characteristics and robust bioactivity profiles to have appeared as promising synthetic targets, as these heterocyclic hybrids serve as very useful molecular architectures for the investigation of pharmacophore space by making use of diverse oriented synthesis that in turn results in the development of new drug entrants [19,20,21,22,23,24]. The literature reports discovered that the presence of α,β-unsaturated ketone moiety in a piperidone molecular unit is an important functionality for the activity of piperidone derivatives. Besides, these compounds are investigated to be associated with both fluorescence and antitumor capacity and thus can be potential for cancer cell studies towards the development of novel therapeutic pathways involving fluorescent drugs [25]. However, the literature reports provide the information that the combination of these biologically important structural units in a single molecular framework has not been widely studied. Therefore, based on the excellent biological profiles of spiropyrrolidine oxindoles, piperidone, and α,β-unsaturated ketones, we wondered the molecular hybridization of these structural subunits in a single molecule that might create novel anticancer drugs. Hence, in this research, we aimed to discuss our efforts in the design and development of more potent analogues for the cancer chemotherapy.

## 2. Results and Discussion

### 2.1. Chemistry

**Scheme 1:**
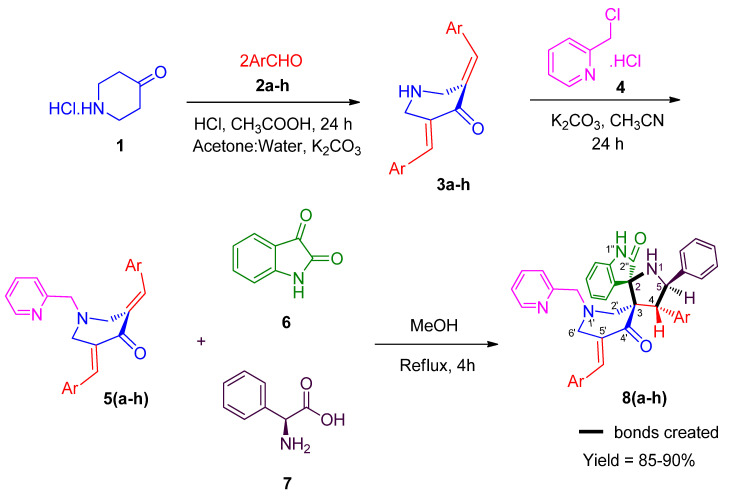
Synthesis of spirooxindole-pyrrolidine molecular scaffolds **8**(**a**–**h**).

In our initial attempt, the synthesis of *N*-unsubstituted bisarylmethylidene-tetrahydropyridinones **3** (**a–h**) was performed according to the literature report as provided by Dimmock et al. [26]. With the compounds **3** (**a**–**h**) in hand, the dipolarophiles and *N*-substituted bisarylmethylidene-tetrahydropyridinones **5** (**a**–**h**) required for the present study were synthesized in a 85–90% yield range through the alkylation of **3** (**a**–**h**) with 2-(chloromethyl)pyridine hydrochloride in the presence of K_2_CO_3_ (Scheme 1) [27,28]. The azomethine ylide was generated in situ from isatin and phenylglycine via decarboxylative condensation. Our approach to the synthesis of spirooxindole molecular scaffold employing isatin **6** and (L)-Phenylglycine **7** was based on a multicomponent reaction strategy involving the 1,3-dipolar cycloaddition reaction between *N*-pyridinylmethyl-bisarylmethylidenepyridinones **5** (**a**–**h**) and azomethine ylide generated in situ from **6** and **7** as outlined in Scheme 1.

Initially, the reaction optimization for this one-pot (3+2) cycloaddition reaction was done by performing the reaction of an equimolar mixture of **5e**, **6**, and **7** under reflux in different solvents, including ethanol, methanol, dioxane, methanol/dioxane, and acetonitrile. The reaction progress was observed by thin layer chromatography, after the completion of the reaction, as evidenced by the disappearance of the substrates and the formation of a sole reaction product by thin-layer chromatography (TLC) analysis. Fifty milliliters of ice-cold water were added to the reaction mixture, and the precipitated solid was filtered, washed repeatedly with water and then dried under vacuum to obtain the cycloadduct **8e**. The obtained spirooxindole-pyrrolidine heterocyclic hybrid was purified either by crystallization using acetonitrile or by column chromatography employing hexane-ethyl acetate (*v/v*: 3:2) as an eluent. Among the solvents used, methanol was found to be the optimal solvent with respect to yield (88%) and reaction time (2 h). Hence, all the subsequent reactions starting with different aryl ring-substituted dipolarophiles **5** (**b**–**h**) were performed under this optimized reaction condition. All these reactions furnished the corresponding spirooxindole-pyrrolidine heterocyclic hybrids **8** (**a**–**d** and **f**–**h**) in good to excellent yields (85–90%).

This cycloaddition reaction proceeded regio- and stereoselectively, as only one diastereoisomeric cycloadduct is formed despite the presence of multiple stereocenters and afforded the compounds **8** (**a**–**h**) in good yields. The spirooxindole-pyrrolidine molecular scaffolds were obtained through a process which involved the formation of two C–C bonds, one C–N bond, and four contiguous stereocenters.

**Figure 1:**
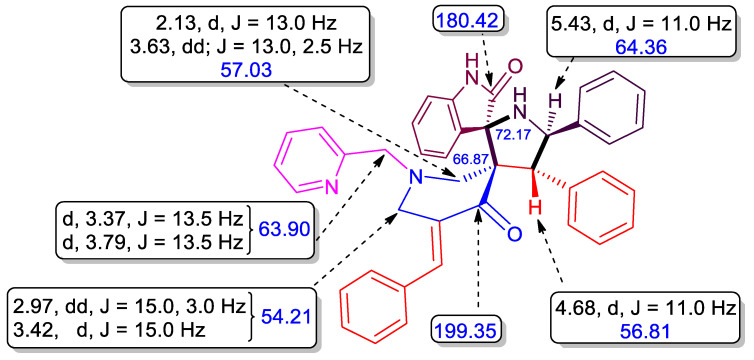
Selected ^1^H and ^13^C NMR chemical shifts of **8e.**

The spirooxindole-pyrrolidine molecular scaffolds **8** were studied thoroughly by making use of various instrumental techniques involving FT-IR, one-dimensional (1D) and two-dimensional (2D) NMR spectroscopy, and mass spectrometry (Figure 1 and †Electronic Supplementary Information). In the FT-IR spectrum of 8e, the noticeable infrared absorption peaks at υ_max_ of 2360, 2341, 1698, and 1597 cm^–1^ were referred to N–H, C=O, and C=C groups, respectively. In the ^1^H NMR spectrum, the H-4 proton furnished a doublet at 4.68 ppm (*J* = 11.0 Hz). This is further established by its Heteronuclear Multiple Bond Correlation (HMBC) with the carbonyl carbon C-4′ at 199.35 ppm. The H,H-Correlation Spectroscopy (H,H-COSY) of H-4 proton allocated the doublet at 5.43 ppm (*J* = 11.0 Hz) to its coupling partner H-5. The C,H-COSY correlation of H-4 and H-5 with the carbon signals at 56.81 ppm and 64.36 ppm assigned these peaks to C-4 and C-5, respectively. Further, the HMBC of H-4 allocated the carbon signal at 57.03 ppm to C-2′. The C,H-COSY correlation of C-2′ assigned the doublet at 2.13 ppm (*J =* 13.0 Hz) and the doublet of doublets at 3.63 ppm (*J =* 13.0 and 2.5 Hz) to 2′-CH_2_. The doublet of doublets at 2.97 ppm (*J =* 15.0 and 3.0 Hz) and the doublet at 3.42 ppm (*J =* 15.0 Hz) were assigned to 6′-CH_2_. The remaining doublets at 3.37 ppm and 3.79 ppm (*J =* 13.5 Hz) were due to 7′-CH_2_. The singlets at 2.26 and 2.27 ppm were due to the two -H_3_ group substituted at the aromatic ring, while the aromatic ring protons appeared as doublets and multiplets around 6.61 to 8.45 ppm. The carbon signals at 54.21 and 63.90 ppm were allocated to C-6′ and C-7′, respectively, whereas the signals at 66.87 ppm and 72.17 ppm were due to the spiro carbons C-3 and C-2, respectively. The oxindole carbonyl carbon appears at 180.42 ppm. The selected ^1^H and ^13^C chemical shifts of 8e are shown in Figure 1. A peak at 603 [M^+^] in the mass spectrum of 8a confirmed the structure deduced from spectroscopic studies.

**Scheme 2:**
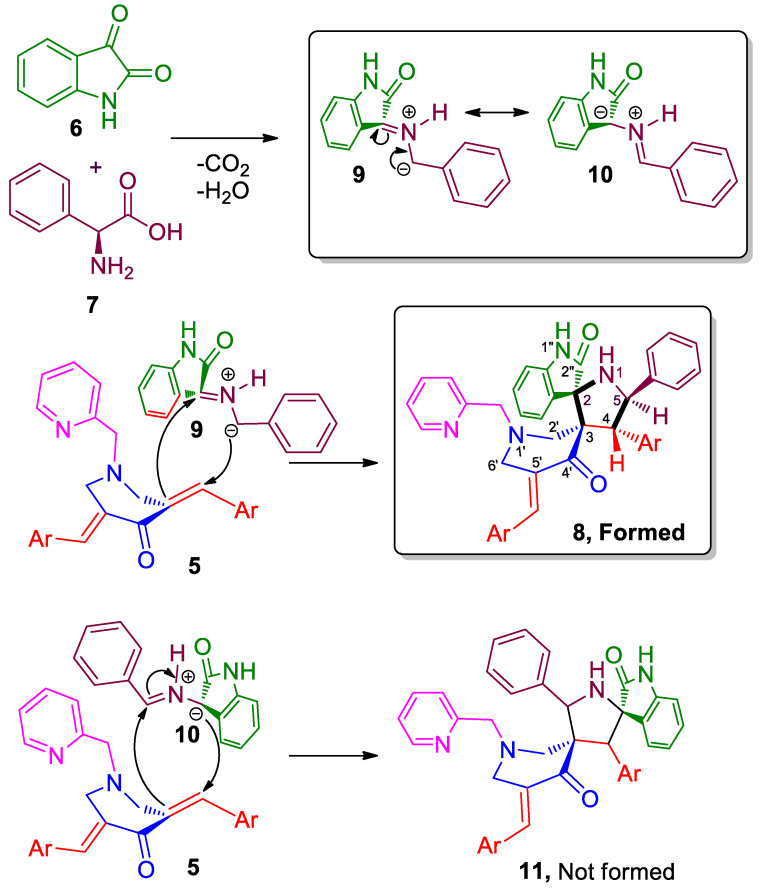
Schematic representation of the plausible mechanism for the region-selective formation of spirooxindole-pyrrolidine molecular scaffolds **8.**

A probable mechanism accounting for the formation of molecular scaffolds **8**(**a**–**h**) is shown in Scheme 2. Presumably, the reaction of isatin and (L)-Phenylglycine via decarboxylative condensation afforded the azomethine ylide which may exist in the resonating forms **9** and **10**. Then, the regioselective addition of ylide **9** to one of the exocyclic C=C bonds of **5** (**a**–**h**)**,** with the addition of nucleophilic carbon of the azomethine ylide to the more electron-deficient β-carbon of the dipolarophile to afford the cycloadducts **8** (**a**–**h**). The other possible regioisomer **11** formed by the addition of azomethine ylide **10** to the dipolarophile was not observed even in traces. The nonformation of this regioisomer may be attributed to the existence of steric hindrance experienced by the aryl and oxindole rings.

### 2.2. Biology

**Figure 2:**
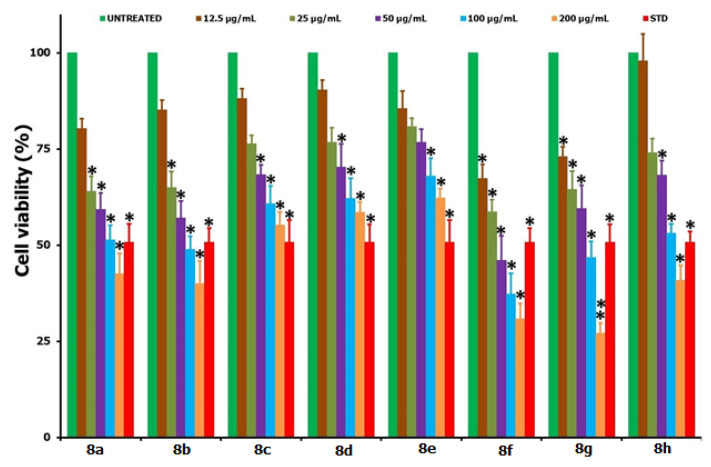
Comparison of cell viability results in vitro from the MTT assay against the HepG2 cells when treated with the tested compounds **8**(**a**–**h**) with a range of concentrations (12.5–200 µg/mL; 24 h period). From the figure, Standard (STD) stands for melphalan (15 µg/mL); * and ** relate to the significant (*p* < 0.05) and highly significant (*p* < 0.01) values as compared against those obtained with the untreated measurements.

**Figure 3:**
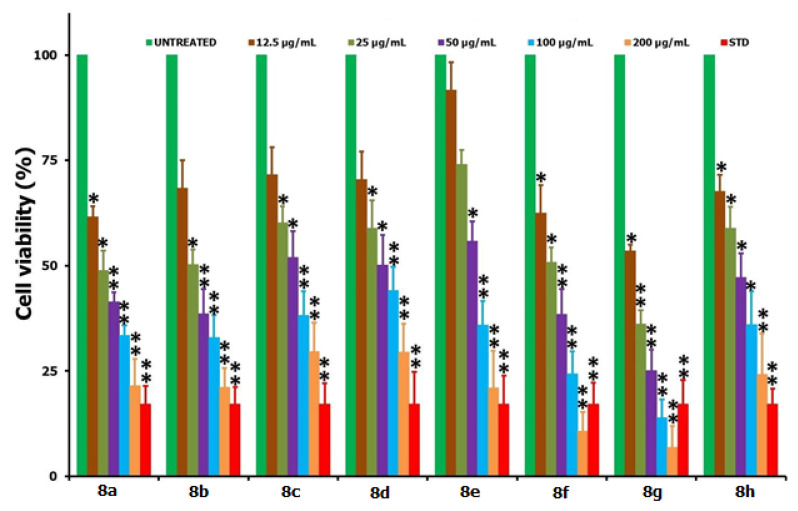
Cell viability comparison of MTT assay results of HepG2 cells from the treatment of compounds **8** (**a**–**h**) over a range of concentration (12.5–200 µg/mL; 48 h period). From the figure, STD stands for melphalan (15 µg/mL); * and ** relate to the significant (*p* < 0.05) and highly significant (*p* < 0.01) values as compared to those obtained with the untreated measurements.

The in vitro studies of cell viability due to the action of the tested compounds **8** (**a**–**h**) at the treatments in the concentration range of 12.5–200 µg/mL over two different time periods of 24 and 48 h to HepG2 cancer cells are shown in Figure 2 and Figure 3. It can be noted from the results analysis that all the tested compounds maintained certain levels of activity against the cancer cells and this activity was increased with regards to an increased concentration and an increased incubation period of the tested compounds, as against the positive control of melphalan (15 µg/mL) and the negative control of the cells having no treatment. Table 1 indicates the concentration required for 50% of cell death (IC_50_) values following the treatment of compounds **8** (**a**–**h**) for the HepG2 cancer cells for 24 and 48 h periods and reveals the highest activities for the two compounds **8f** and **8g** and the least activity for the compound **8e**. Among the two most active compounds of **8f** and **8g**, whether one took the lead over the other depended upon the incubation time. That is, under the 24 h incubation time, the compound **8f** is most active, as we observed the IC_50_ value of approximately 43.46 µg/mL, while at the 48 h incubation period, the compound **8g** has an IC_50_ value of approximately 12.79 µg/mL. Thus, based on the MTT anticancer activity assay, the observed IC_50_ value order during the 24 h incubation period followed the order of **8f** > **8g** > **8b** > **8a** > **8h** > **8c** > **8d** > **8e,** while for the 48 h period it is **8g** > **8f** > **8a** > **8b** > **8h** > **8c** > **8d** > **8e**.

**Figure 4:**
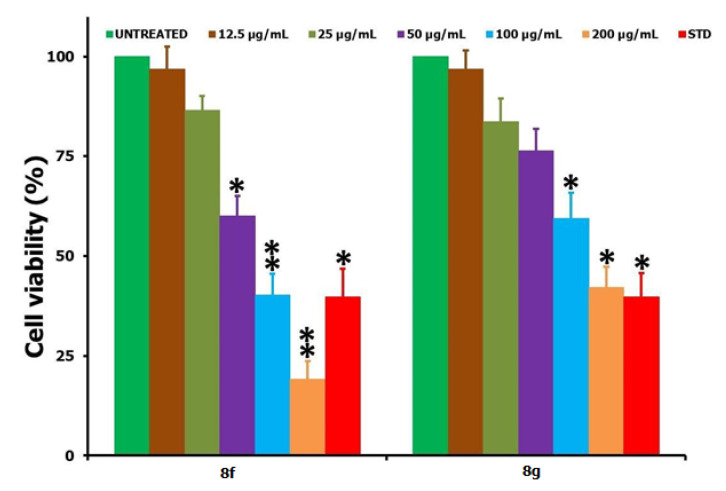
Comparison of MTT assay results of L929 noncancer cells following the incubation of two compounds **8f** and **8g** with a range of concentrations (12.5–200 µg/mL) over a 48 h period. STD stands for melphalan of 15 µg/mL; * and ** refer to the significant (*p* < 0.05) and highly significant (*p* < 0.01) values as compared to those obtained with the untreated controls.

To test the behavior of our synthesized compounds towards the noncancer cells, the two most active compounds, **8f** and **8g** were tested against the L929 cells over a concentration range of 12.5–200 µg/mL for the highest incubation period of 48 h (as shown in Figure 4 and the values tabulated in Table 1). From the analysis of the experimental results, it can be noted that the two compounds exhibited an approximately similar activity and the considerable activity was observed only at and above the concentration of 100 µg/mL. The IC_50_ values of the compounds **8f** and **8g** were measured to be 72.12 and 153.89 µg/mL, respectively, and these values were higher as compared to the IC_50_ values observed for the same compounds but with the cancer cells (HepG2). This indicated that the controlling of cancer cells by the treatment of compounds **8f** and **8g** and with negligible damage to the noncancer cells can be possible and this is one of the important criteria in cancer chemotherapy. In addition, since our aim was to examine the probable mechanism of cell death produced by the most active molecular scaffold, we selected the compound **8g** to perform the other molecular biology studies which required only the least concentration for the observation of cell death.

**Figure 5:**
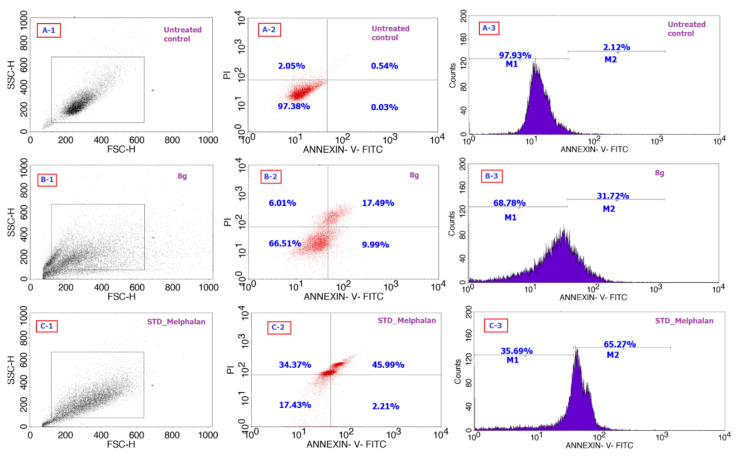
Apoptosis assay comparison of the fluorescence assay results following the treatment of HepG2 cells with that of the tested compound **8g** (**B**). The results were compared with those of melphalan used as the positive control (**C**) and no cell treatment used as the negative control (**A**) over a 48 h period.

To understand the cell death mechanism followed by the treatment of compound **8g**, the apoptosis, reactive oxygen species (ROS), and caspase activity assays were performed, and the obtained results were compared with those of the positive control (melphalan; 15 µg/mL) and the negative control of the cells without any treatment. Figure 5 shows the flow cytometry provided apoptosis assay results of the HepG2 cells due the treatment of the compound **8g** (at IC_50_ concentration.) and the positive control (melphalan) and the negative control of the cells (no treatment). The results of A-2, B-2, and C-2 provided in Figure 5 represented the mixed fluorescence intensities from the cells treated with propidium iodide (PI), and Annexin V-fluorescein isothiocyanate (Annexin V-FITC), the cells treated with the compound **8g**, and melphalan-treated cells, respectively. From the compound **8g**-treated cells, we observed that about 66.5% cells were live, 10% were apoptotic, and 17.5% were late apoptotic/early necrotic (Figure 5(B-2)); these values were far better than those of the melphalan-treated cells where we observed only 17.4% of live cells, 2.2% of apoptotic, and 46% of late apoptotic/early necrotic cells (Figure 5(C-2)). In addition, M1 and M2 shown in Figure 5 (A-3), 5(B-3), and 5(C-3) corresponded to the fluorescence intensities generated from the Annexin V-FITC dye by the viable and apoptotically dying cells, respectively. We observed from the Figure 5 that about 31.7% of the compound **8g**-treated cells were apoptotic and 68.7% of these cells were live. However, 65.2% of the melphalan-treated cells were apoptotic, and 35.6% of those cells were live. Thus, from the analysis of fluorescence intensity results by the PI and Annexin V-FITC, it can be indicated that apoptosis is the dominating mechanism for the observed loss of cell viability in the case of the HepG2 cancer cells.

**Figure 6:**
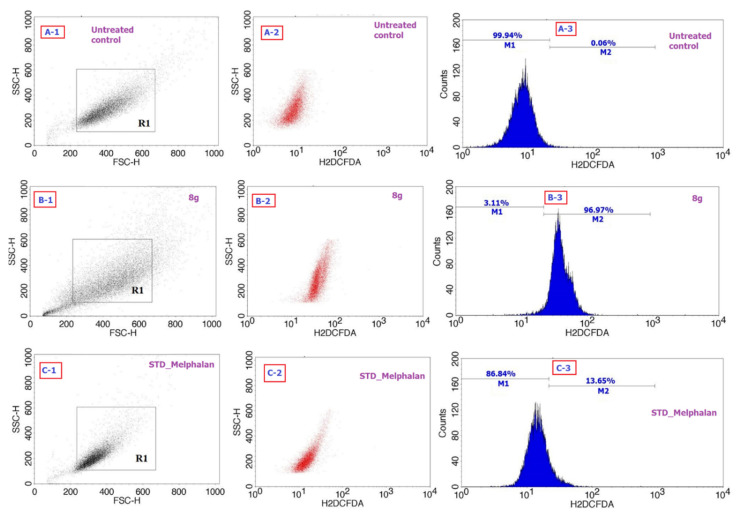
Reactive oxygen species (ROS) generation as a measure of fluorescence intensity in the HepG2 cells following the negative control having no cell treatment (**A**), the treatment of compound **8g** (**B**), and the positive control melphalan (**C**) over a 48 h period.

Figure 6 shows the flow cytometric analysis towards the measurement of ROS generated following the treatment of the compound **8g** (at IC_50_ conc.) to the HepG2 cells when compared with those of the positive control (melphalan) and the negative control (cells of no treatment). From the analysis of results, it can be observed that about 97% of apoptotic cells generated the ROS in the case of the compound **8g** treated cells (Figure 6 (B-3)) and this value was far higher than that of the positive control (melphalan)-treated cells (only 13.6% cells were apoptotic cells; Figure 6 (C-3)). The observation of such a high value of ROS generation from the compound **8g**-treated cells indicated that most of the apoptotic cells released the ROS during their contact with the tested compound.

**Figure 7:**
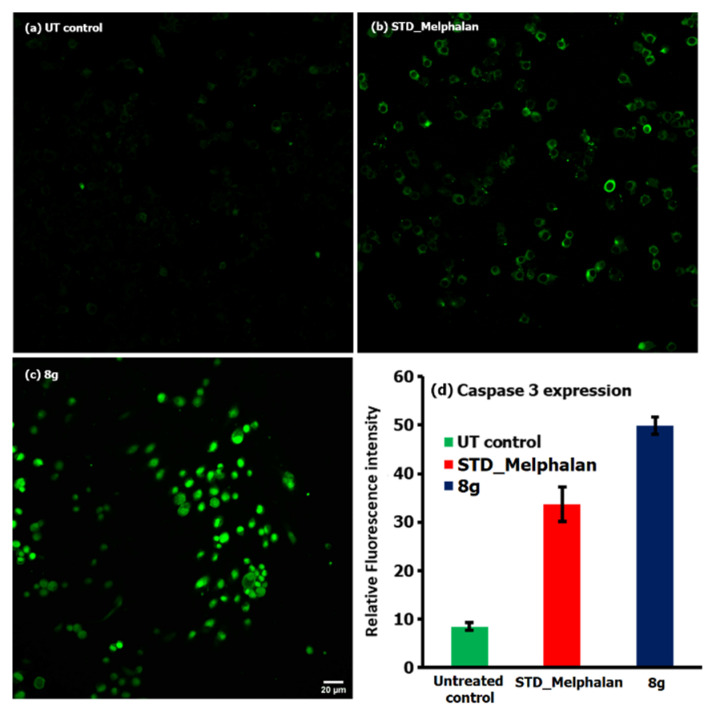
Comparison of caspase-3 activity results provided by the co-incubation of HepG2 cells with the negative control having no cell treatment (**a**), treated with melphalan used as the positive control (**b**), and treated with the compound **8g** (**c**) over a 48 h period. The relative fluorescence intensities of all the treatments were compared in (**d**).

The involvement of caspases by means of caspase-3 activity was tested for the HepG2 cancer cells due to the treatment of the compound **8g** (at IC_50_ conc.), where the results were compared against the untreated and melphalan-treated controls as shown in Figure 7. We observed a very high number of fluorescent cells from the tested compound **8g**-treated cells (Figure 7c) as against those from the positive control (Figure 7b) and the negative control (Figure 7a), and the same trend was quantitatively shown in Figure 7d. This indicated that the HepG2 cells underwent the caspase-3 release before they lost the viability, thereby confirming for the involvement of caspases for the compound **8g**-treated cells.

## 3. Materials and Methods

The detailed materials and methods were provided in Supplementary Materials.

### 3.1. 4-(aryl)-5-phenylpyrrolo(spiro[2.3″]-oxindole)-spiro[3.3′]-5′-(2-arylmethylidene)-1′-N-(pyridinylmethyl)piperidin-4′-one **8** (**a**-**h**)

An equimolar mixture of *N*-substituted bisarylmethylidene-tetrahydropyridinones **5**(**a**–**h**) (0.100 g, 0.27 mmol), isatin **6** (0.040 g, 0.27 mmol), and phenylglycine **7** (0.041 g, 0.27 mmol) were dissolved in methanol (5 mL) and heated under reflux with constant stirring for 4 h. After completing the reaction as evident from TLC, the reaction mixture was transferred into 50 mL of ice-cold water to form a precipitate which was separated out by filtration and washed with water to obtain the product **8**(**a**–**h**) in good yields.

### 3.2. 4,5-Diphenylpyrrolo(spiro[2.3″]-oxindole)-spiro-[3.3′]-5′-(phenylmethylidene)-1′-N-(pyridinylmethyl)piperidin-4′-one (**8a**)

Obtained as a white solid (88%); mp = 219–221 °C; IR (KBr): 2359, 2340, 1698, and 1599 cm^−1^; ^1^H NMR (500 MHz, CDCl_3_): δ_H_ 2.11 (d, 1H, *J =* 12.5 Hz, 2′-CH_2_), 2.51 (brs, NH), 2.96 (dd, 1H, *J =* 15.0, 2.5 Hz, 6′-CH_2_), 3.35 (d, 1H, *J =* 14.0 Hz, 7′-CH_2_), 3.42 (d, 1H, *J =* 14.5 Hz, 6′-CH_2_), 3.65 (dd, 1H, *J =* 13.0, 2.0 Hz, 2′-CH_2_), 3.76 (d, 1H, *J =* 13.5 Hz, 7′-CH_2_), 4.72 (d, 1H, *J =* 10.0 Hz, 4-CH), 5.47 (d, 1H, *J =* 11.0 Hz, 5-CH), 6.63 (d, 1H, *J =* 8.0 Hz, ArH), 6.93–7.09 (m, 7H, ArH), 7.18–7.28 (m, 9H, ArH), 7.43 (d, 2H, *J =* 7.5 Hz, ArH), 7.47 (dd, 1H, *J =* 7.5, 2.0 Hz, ArH), 7.54 (d, 2H, *J =* 7.5 Hz, ArH), 8.26 (s, 1H, NH), 8.42–8.43 (m, 1H, ArH). ^13^C NMR (125 MHz, CDCl_3_): δ_C_ 53.95, 56.95, 57.04, 63.68, 64.23, 67.01, 72.03, 109.11, 122.05, 122.07, 123.06, 126.85, 126.96, 127.52, 127.64, 128.21, 128.28, 128.70, 128.88, 129.01, 129.90, 130.00, 132.91, 134.82, 136.17, 137.31, 137.60, 140.80, 141.51, 148.94, 157.34, 180.53, and 199.33. Mass: 603 [M^+^]. Anal. calc. for C_40_H_34_N_4_O_2_: C, 79.71; H, 5.69; N, 9.30. Found: C, 79.85; H, 5.51; N, 9.44%.

### 3.3. 4-(2-Methylphenyl)-5-phenylpyrrolo(spiro[2.3″]-oxindole)spiro[3.3′]-5′-(2-methylphenyl-methylidene)-1′-N-(pyridinylmethyl)piperidin-4′-one (**8b**)

Obtained as a white solid (90%); mp = 190–193 °C; IR (KBr): 2358, 2337, 1699, and 1597 cm^−1^; ^1^H NMR (500 MHz, CDCl_3_): δ_H_ 2.10 (d, 1H, *J =* 13.0 Hz, 2′-CH_2_), 2.11 (s, 3H, CH_3_), 2.16 (s, 3H, CH_3_), 2.90 (dd, 1H, *J =* 15.0, 3.0 Hz, 6′-CH_2_), 3.24-3.28 (m, 2H, 7′-CH_2_, 6′-CH_2_), 3.51 (dd, 1H, *J =* 13.0, 2.0 Hz, 2′-CH_2_), 3.63 (d, 1H, *J =* 14.0 Hz, 7′-CH_2_), 4.92 (d, 1H, *J =* 10.5 Hz, 4-CH), 5.53 (d, 1H, *J =* 10.0 Hz, 5-CH), 6.68-6.71 (m, 2H, ArH), 6.80 (d, 1H, *J =* 8.0 Hz, ArH), 6.93–7.20 (m, 6H, ArH), 7.23–7.30 (m, 8H, ArH), 7.41–7.62 (m, 4H, ArH), 8.07 (s, 1H, ArH), 8.38–8.39 (m, 1H, ArH). ^13^C NMR (125 MHz, CDCl_3_): δ_C_ 20.13, 21.19, 54.04, 54.64, 58.52, 64.38, 65.11, 66.11, 73.81, 109.34, 122.07, 122.50, 122.60, 125.56, 125.98, 126.70, 127.05, 127.53, 128.25, 128.45, 128.52, 128.83, 128.89, 129.56, 130.21, 130.28, 132.53, 134.01, 136.16, 136.39, 137.45, 137.94, 141.50, 141.89, 149.06, 157.73, 179.23, and 199.89. Mass: 631 [M^+^]. Anal. calc. for C_42_H_38_N_4_O_2_: C, 79.97; H, 6.07; N, 8.88. Found: C, 79.85; H, 6.16; N, 8.97%.

### 3.4. 4-(2-Methoxyphenyl)-5-phenylpyrrolo(spiro[2.3″]-oxindole)spiro[3.3′]-5′-(2-methoxy-phenylmethylidene) 1′-N-(pyridinylmethyl)piperidin-4′-one (**8c**)

Obtained as a reddish brown solid (86%); mp = 170–173 °C; IR (KBr): 2359, 2339, 1697, and 1598 cm^−1^; ^1^H NMR (500 MHz, CDCl_3_): δ_H_ 2.20 (d, 1H, *J =* 12.5 Hz, 2′-CH_2_), 2.95 (dd, 1H, *J =* 15.0, 3.0 Hz, 6′-CH_2_), 3.26 (d, 1H, *J =* 13.5 Hz, 7′-CH_2_), 3.34 (dd, 1H, *J =* 15.0 Hz, 6′-CH_2_), 3.47 (s, 3H, OCH_3_), 3.64 (dd, 1H, *J =* 12.5, 2.5 Hz, 2′-CH_2_), 3.72 (s, 3H, OCH_3_), 3.78 (d, 1H, *J =* 13.5 Hz, 7′-CH_2_), 4.93 (d, 1H, *J =* 10.0 Hz, 4-CH), 5.67 (d, 1H, *J =* 10.5 Hz, 5-CH), 6.65–6.72 (m, 2H, ArH), 6.84 (d, 1H, *J =* 7.5 Hz, ArH), 6.95-7.32 (m, 16H, ArH), 7.40–7.65 (m, 4H, ArH), 8.08 (s, 1H, ArH), 8.39–8.41 (m, 1H, ArH). ^13^C NMR (125 MHz, CDCl_3_): δ_C_ 54.03, 54.72, 55.42, 55.87, 58.57, 64.43, 65.16, 66.24, 73.88, 109.31, 112.98, 122.27, 122.55, 122.64, 125.51, 126.00, 126.73, 127.11, 127.54, 128.29, 128.56, 128.85, 128.93, 129.63, 130.20, 130.38, 132.54, 13408, 136.33, 136.45, 137.41, 140.50, 141.76, 149.09, 157.70, 158.11, 160.98, 179.72, and 199.85. Mass: 663 [M^+^]. Anal. calc. for C_42_H_38_N_4_O_4_: C, 76.11; H, 5.78; N, 8.45. Found: C, 76.30; H, 5.90; N, 8.31%.

### 3.5. 4-(3-Nitrophenyl)-5-phenylpyrrolo(spiro[2.3″]-oxindole)spiro[3.3′]-5′-(3-nitrophenyl methylidene)-1′-N-(pyridinylmethyl)piperidin-4′-one (**8d**)

Obtained as a white solid (87%); mp = 193–196 °C; IR (KBr): 2358, 2336, 1698, 1597 cm^−1^; ^1^H NMR (500 MHz, CDCl_3_): δ_H_ 1.99 (d, 1H, *J =* 13.0 Hz, 2′-CH_2_), 2.99 (dd, 1H, *J =* 15.0, 2.5 Hz, 6′-CH_2_), 3.34 (d, 1H, *J =* 15.0 Hz, 7′-CH_2_), 3.40 (d, 1H, *J =* 15.0 Hz, 6′-CH_2_), 3.70 (dd, 1H, *J =* 13.0, 3.0 Hz, 2′-CH_2_), 3.77-3.92 (m, 1H, 7′-CH_2_), 4.77 (d, 1H, *J =* 10.5 Hz, 4-CH), 5.49 (d, 1H, *J =* 11.0 Hz, 5-CH), 6.65 (d, 1H, *J =* 7.5 Hz, ArH), 6.96–7.06 (m, 1H, ArH), 7.10–7.27 (m, 11H, ArH), 7.40–7.48 (m, 1H, ArH), 7.52–7.82 (m, 6H, ArH), 8.05 (d, 1H, *J =* 8.0 Hz, ArH), 8.20 (d, 1H, *J* = 8.0 Hz, ArH), 8.24 (s, 1H, NH), 8.48–8.50 (m, 1H, ArH). ^13^C NMR (125 MHz, CDCl_3_): δ_C_ 54.41, 56.21, 56.48, 63.29, 64.32, 67.10, 71.51, 109.51, 122.58, 122.66, 123.29, 126.92, 127.03, 127.68, 128.20, 128.43, 128.65, 129.43, 129.77, 130.28, 132.62, 134.21, 134.65, 135.35, 135.46, 136.16, 136.34, 136.61, 138.37, 139.42, 139.98, 140.09, 141.68, 148.09, 149.54, 156.49, 181.90, and 198.37. Mass: 693 [M^+^]. Anal. calc. for C_40_H_32_N_6_O_6_: C, 69.35; H, 4.66; N, 12.13. Found: C, 69.46; H, 4.74; N, 12.25%.

### 3.6. 4-(4-Methylphenyl)-5-phenylpyrrolo(spiro[2.3″]-oxindole)spiro[3.3′]-5′-(4-methylphenyl-methylidene)-1′-N-(pyridinylmethyl)piperidin-4′-one (**8e**)

Obtained as a white solid (90%); mp = 195–198 °C; IR (KBr): 2360, 2341, 1698, and 1597 cm^−1^; ^1^H NMR (500 MHz, CDCl_3_): δ_H_ 2.13 (d, 1H, *J =* 13.0 Hz, 2′-CH_2_), 2.26 (s, 3H, CH_3_), 2.27 (s, 3H, CH_3_), 2.97 (dd, 1H, *J =* 15.0, 3.0 Hz, 6′-CH_2_), 3.37 (d, 1H, *J =* 13.5 Hz, 7′-CH_2_), 3.42 (d, 1H, *J =* 15.0 Hz, 6′-CH_2_), 3.63 (dd, 1H, *J =* 13.0, 2.5 Hz, 2′-CH_2_), 3.79 (d, 1H, *J =* 13.5 Hz, 7′-CH_2_), 4.68 (d, 1H, *J =* 11.0 Hz, 4-CH), 5.43 (d, 1H, *J =* 11.0 Hz, 5-CH), 6.62 (d, 1H, *J =* 7.5 Hz, ArH), 6.87 (d, 2H, *J =* 8.5 Hz, ArH), 6.91–7.10 (m, 9H, ArH), 7.15–7.19 (m, 1H, ArH), 7.20–7.26 (m, 3H, ArH), 7.31 (d, 2H, *J =* 8.0 Hz, ArH), 7.46–7.50 (m, 1H, ArH), 7.53–7.55 (m, 2H, ArH), 8.11 (s, 1H, NH), 8.44–8.45 (m, 1H, ArH). ^13^C NMR (125 MHz, CDCl_3_): δ_C_ 21.07, 21.30, 54.21, 56.81, 57.03, 63.90, 64.36, 66.87, 72.17, 109.04, 122.03, 122.05, 123.05, 126.94, 127.46, 127.68, 128.27, 128.80, 128.95, 129.02, 129.08, 129.74, 130.27, 131.97, 132.06, 134.22, 136.20, 136.30, 137.73, 139.14, 140.94, 141.47, 148.97, 157.54, 180.42, 199.35. Mass: 631 [M^+^]. Anal. calc. for C_42_H_38_N_4_O_2_: C, 79.97; H, 6.07; N, 8.88. Found: C, 79.81; H, 6.21; N, 8.75%.

### 3.7. 4-(4-Chlorophenyl)-5-phenylpyrrolo(spiro[2.3″]-oxindole)spiro[3.3′]-5′-(4-chlorophenyl-methylidene)-1′-N-(pyridinylmethyl)piperidin-4′-one (**8f**)

Obtained as a white solid (87%); mp = 198–202 °C; IR (KBr): 2359, 2340, 1698, and 1597 cm^−1^; ^1^H NMR (500 MHz, CDCl_3_): δ_H_ 2.06 (d, 1H, *J =* 12.5 Hz, 2′-CH_2_), 2.95 (dd, 1H, *J =* 15.0, 3.0 Hz, 6′-CH_2_), 3.34–3.40 (m, 2H, 7′-CH_2_, 6′-CH_2_), 3.62 (dd, 1H, *J =* 13.0, 2.5 Hz, 2′-CH_2_), 3.80 (d, 1H, *J =* 13.0 Hz, 7′-CH_2_), 4.65 (d, 1H, *J =* 10.5 Hz, 4-CH), 5.40 (d, 1H, *J =* 10.5 Hz, 5-CH), 6.60 (d, 1H, *J =* 7.5 Hz, ArH), 6.84 (d, 2H, *J =* 8.5 Hz, ArH), 6.92–6.99 (m, 2H, ArH), 7.05–7.11 (m, 2H, ArH), 7.16–7.28 (m, 9H, ArH), 7.35 (d, 1H, *J =* 8.0 Hz, ArH), 7.50–7.53 (m, 4H, ArH), 7.83 (s, 1H, NH), 8.47–8.48 (m, 1H, ArH). ^13^C NMR (125 MHz, CDCl_3_): δ_C_ 53.88, 56.23, 56.93, 63.70, 64.36, 67.00, 71.90, 109.23, 122.14, 122.38, 123.26, 127.09, 127.68, 127.83, 128.48, 128.56, 128.63, 128.92, 129.09, 131.27, 132.84, 133.25, 133.34, 134.92, 135.86, 136.33, 136.38, 140.54, 141.55, 149.29, 157.21, 180.40, and 198.97. Mass: 671 [M^+^]. Anal. calc. for C_40_H_32_Cl_2_N_4_O_2_: C, 71.53; H, 4.80; N, 8.34. Found: C, 71.74; H, 4.91; N, 8.25%.

### 3.8. 4-(4-Fluorophenyl)-5-phenylpyrrolo(spiro[2.3″]-oxindole)spiro[3.3′]-5′-(4-fluorophenyl-methylidene)-1′-N-(pyridinylmethyl)piperidin-4′-one (**8g**)

Obtained as a white solid (85%); mp = 164–166 °C; IR (KBr): 2360, 2337, 1698, and 1597 cm^−1^; ^1^H NMR (500 MHz, CDCl_3_): δ_H_ 2.07 (d, 1H, *J =* 12.0 Hz, 2′-CH_2_), 2.98 (d, 1H, *J =* 15.0 Hz, 6′-CH_2_), 3.32-3.34 (m, 2H, 7′-CH_2_, 6′-CH_2_), 3.60 (dd, 1H, *J =* 15.0, 5.0 Hz, 2′-CH_2_), 3.80 (d, 1H, *J =* 12.5 Hz, 7′-CH_2_), 4.66 (d, 1H, *J =* 10.0 Hz, 4-CH), 5.39 (d, 1H, *J =* 11.0 Hz, 5-CH), 6.61 (d, 1H, *J =* 7.0 Hz, ArH), 6.88–7.10 (m, 9H, ArH), 7.20–7.41 (m, 7H, ArH), 7.50–7.53 (m, 3H, ArH), 7.75 (s, 1H, NH), 8.44–8.48 (m, 1H, ArH). ^13^C NMR (125 MHz, CDCl_3_): δ_C_ 54.06, 56.99, 57.08, 63.94, 64.31, 66.82, 72.10, 109.11, 115.17, 115.35, 122.09, 122.25, 123.07, 126.91, 127.45, 128.31, 128.94, 129.73, 130.28, 131.94, 132.16, 134.26, 136.24, 136.39, 137.71, 140.91, 141.48, 148.93, 157.55, 158.24, 160.17, 180.39, and 199.28. Mass: 631 [M^+^]. Anal. calc. for C_40_H_32_F_2_N_4_O_2_: C, 75.22; H, 5.05; N, 8.77. Found: C, 75.38; H, 5.21; N, 8.65%.

### 3.9. 4-(2,4-Dichlorophenyl)-5-phenylpyrrolo(spiro[2.3″]-oxindole)spiro[3.3′]-5′-(2,4-dichloro-phenylmethylidene)-1′-N-(pyridinylmethyl)piperidin-4′-one (**8h**)

Obtained as a yellow solid (89%); mp = 125–128 °C; IR (KBr): 2359, 2340, 1699, and 1595 cm^−1^; ^1^H NMR (500 MHz, CDCl_3_): δ_H_ 2.21 (d, 1H, *J =* 13.0 Hz, 2′-CH_2_), 2.98 (d, 1H, *J =* 14.5 Hz, 6′-CH_2_), 3.21 (d, 1H, *J =* 14.0 Hz, 7′-CH_2_), 3.29 (d, 1H, *J =* 14.5 Hz, 6′-CH_2_), 3.61 (d, 1H, *J =* 14.0 Hz, 2′-CH_2_), 3.71-3.76 (m, 1H, 7′-CH_2_), 5.07 (d, 1H, *J =* 9.5 Hz, 4-CH), 5.52 (d, 1H, *J =* 9.5 Hz, 5-CH), 6.70 (t, 1H, *J =* 7.5 Hz, ArH), 6.76 (d, 1H, *J =* 8.0 Hz, ArH), 6.93–7.34 (m, 11H, ArH), 7.41–7.48 (m, 3H, ArH), 7.60 (d, 1H, *J* = 7.5 Hz, ArH), 7.74 (s, 1H, NH), 7.88 (m, 1H, ArH), 8.10 (d, 1H, J = 8.5 Hz, ArH), 8.41–8.42 (m, 1H, *J* = 4.5 Hz, ArH). ^13^C NMR (125 MHz, CDCl_3_): δ_C_ 54.23, 54.66, 57.87, 64.30, 64.50, 65.53, 74.55, 109.39, 122.24, 122.43, 122.97, 126.83, 126.94, 127.34, 127.71, 128.04, 128.73, 128.98, 129.25, 129.83, 129.90, 130.00, 130.49, 130.98, 131.69, 133.27, 133.70, 134.10, 134.81, 135.99, 136.32, 140.45, 141.61, 149.16, 157.41, 166.79, 178.05, and 198.82. Mass: 741 [M^+^]. Anal. calc. for C_40_H_30_Cl_4_N_4_O_2_: C, 64.88; H, 4.08; N, 7.57. Found: C, 64.79; H, 4.21; N, 7.42%.

## 4. Conclusions

An efficient synthesis of a series of novel spirooxindole-pyrrolidine heterocyclic molecular scaffolds in good to excellent yields has been achieved using a one-pot three-component (3+2) cycloaddition reaction of azomethine ylide generated in situ from isatin and phenylglycine with a new class of functionalized dipolarophiles. The structure of all the new compounds were elucidated by FT-IR, NMR spectroscopy and mass spectrometric data. The synthesized spirooxindole heterocyclic hybrids were tested to investigate the extent of anticancer activities against the HepG2 cancer cells in vitro (up to 200 µg/mL; 24–48 h time period). The comparative analysis of the obtained results with those of the positive control of melphalan (15 µg/mL) and the negative control of no cell treatments showed that the highest activity was obtained for the compound **8g** over a 48 h period (i.e., obtained the least IC_50_ value of only 12.7 µg/mL). From the further investigation of the mechanism of toxicology, we found that the HepG2 cells experienced the apoptotic pathway with the induction of ROS generation and the involvement of caspases. This type of cell death mechanism is a potential pathway for the treatment of cancer cells, as noncancer cells do not have to scarify and full control over diseased cells can be possible.

## Figures and Tables

**Table 1:** Comparison of the concentration required for 50% of cell death (IC_50_) of the tested compounds **8**(**a**–**h**) following the treatments to the HepG2 and L929 cells over 24 and 48 h incubation periods.

Entry	Compound	IC_50_ Values (µg/mL) HepG2 Cells		IC_50_ Values (µg/mL) L929 Cells
		24 h Treatment	48 h Treatment	48 h Treatment
1	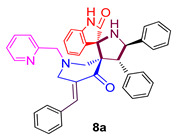	105.78 ± 2.1	27.43 ± 4.1	NA
2	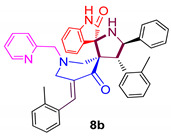	91.5 ± 2.1	31.21 ± 5.3	NA
3	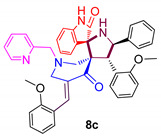	274.61 ± 2.1	52.14 ± 6.7	NA
4	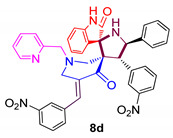	352.86 ± 2.1	53.04 ± 5.5	NA
5	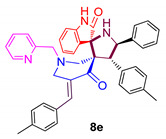	897.42 ± 2.1	62.25 ± 5.8	NA
6	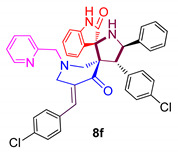	43.46 ± 2.1	25.82 ± 3.2	72.12 ± 3.3
7	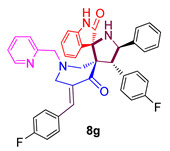	65.18 ± 2.1	12.79 ± 2.6	153.89 ± 4.2
8	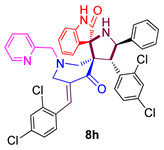	120.76 ± 2.1	41.32 ± 3.2	NA

## Supplementary Materials

The following are available online. Detailed methodology, NMR, IR and mass spectra of a representative compound.
