# Supplementary material for: A One-Pot Three-Component Synthesis and Investigation of the In Vitro Mechanistic Anticancer Activity of Highly Functionalized Spirooxindole-Pyrrolidine Heterocyclic Hybrids
Source: Molecules. 2020 Nov 27;25(23):5581. doi: 10.3390/molecules25235581 (PMC7730040; doi:10.3390/molecules25235581)
Supplement: Supplementary file 1 [file molecules-25-05581-s001.pdf]

Supplementary Material

Experimental section

Materials and methods

Chemistry

All reagents and solvents used in the present study were procured from commercial suppliers and used without further purification. The reactions were monitored by TLC on silica gel. Melting points were measured using open capillary tubes and were uncorrected. ^{1}H, ^{13}C, and two-dimensional NMR spectra were recorded on a Jeol 500 MHz instrument in $CDCl_3$ using Tetramethylsilane (TMS) as an internal standard. Standard Jeol software was used throughout the process. Chemical shifts were given in parts per million (δ-scale), and the coupling constants were given in Hertz. FT-IR spectra were recorded on a Perkin Elmer system 2000 FT-IR instrument (KBr). Mass spectra were recorded on a DART-ToF-MS mass spectrometer.

Biology

Cell culture and maintenance

For the testing of the anticancer efficacy of our synthesized compounds, two different cell lines, one cancerous cell line and the other noncancerous cell line, were selected. The cancer cell line included HepG2 cells of human hepatic cells, and the selected noncancer cells were L929 cells of mouse subcutaneous connective tissue, obtained from NCCS, Pune, India. Both the cell lines were cultured individually in Eagle's Minimum Essential Medium (EMEM) supplemented with 10% Fetal Bovine Serum (FBS) that contained antibiotics, 100 U/mL streptomycin, and 100 U/mL penicillin. For the culture, the cells were suspended in cell culture flasks and incubated at 37 °C in 5% CO_2 humidified atmosphere. The cells were extracted when the growth reached 80% confluency. The cell counting was done with an aid of a hemocytometer, and the corresponding assays were performed after calculating the cell viability and seeding.

MTT cytotoxicity assay

To understand the anticancer cancer efficacy of the synthesized cage like compounds **8(a–h)**, the MTT assay was performed and was based on the reduction of MTT dye by the cells. The HepG2 cells having the suspension of 2×10^4 cells per well, and 200 μL of EMEM were added to 96-well plates. The cell growth was allowed for 12 h. After the period, different concentrations of the test compounds in the range of 0 to 200 μM were added to the wells and incubated for another 24 or 48 h in 5% CO_2 atmosphere. Then, the medium was removed. A fresh medium containing 2% FBS was added and incubated for another 2 h, and 20 μL (5 mg/mL) of MTT reagent was added to the cells and incubated in CO_2 atmospheric air for another 2 h. At the end of incubation, the formazan crystals formed were dissolved in 100 μL of dimethylsufoxide (DMSO), and the absorbance was measured at 570 nm using an ELISA microplate reader. The cells without any testing sample treatment were taken as the negative control with 100 % viability and melphalan of a 15 μM concentration was taken as the positive control where the % (percentage) of cell viability was calculated using the following equation:

$$\% \, viability = \left(\frac{Mean\ absorbance\ of\ test\ sample}{Mean\ absorbance\ of\ negative\ control} \right) \mathrm{x}\ 100$$

Similarly, the IC_{50} values were determined for all the tested samples. Based on the MTT assay results and the IC_{50} values, the highly effective samples (**8f** and **8g**) were determined, and the cell

viability studies were performed using the noncancer L929 cell line so as to understand those active samples behavior in the healthy normal cells.

Apoptosis assay

The apoptotic assay was carried out on the HepG2 cells using the tested sample of **8g** with an Annexin V-FITC kit from BD Biosciences (catalog no. 556547). For the assay, the cells at a density of 1×10^6 cells/well were plated and allowed to settle for 12 h. After the period, the wells were treated with the IC_{50} concentrations of the tested compound (**8g**) and the positive control of melphalan and incubated for another 48 h. The FITC-conjugated annexin V and PI fluorescence intensities developed in the cells were examined by the flow cytometry. The cultured test materials were then collected, washed with PBS buffer and resuspended in Annexin V-FITC buffer. The suspension was incubated at 25 °C for 10 min in dark with 5 μL of Annexin V-FITC supplied by the manufacturer. The cells were collected by centrifugation and resuspended in Annexin V-FITC buffer, and 5 μL of PI was added in an ice bath. The cells were analyzed by a flow cytometry instrument that used the Cell Quest software (Becton Dickinson Biosciences). Each experiment was repeated thrice for the accuracy, and the average of all the individual experiments was taken as the final where the data shown is the mean ± SD (n=3).

Oxidative stress assay

The generation of oxidative stress due to the treatment of the compound **8g** in the HepG2 cells was studied by analyzing the ROS formed. For the testing, cells at a density of 1×10^5 cells/well were first seeded in 6-well plates, allowed to adhere, and when the cell growth reached its 80% confluence, they were treated with the IC_{50} of the test compounds (**8g** and melphalan), and incubated for another 48 h. After the period, the medium was removed, washed the cells with D-PBS twice and incubated with 10 μM of H2DFCDA (provided by the manufacturer, Invitrogen, catalog number: D399) in dark conditions at room temperature for 30 min. All the protocols were in accordance with the manufacturer's instructions, i.e., the washing of cells again with D-PBS twice, the collection of the pellet following the centrifugation, the resuspension of the pellet in 500 μL of D-PBS, and further analysis of cells by BD FACS Calibur. The compound **8g** results were compared with that of the melphalan-treated cells and the cells without any treatment.

Caspase-3 expression assay

As similar to the earlier assays, the HepG2 cells at a density of 1×10^5 cells/well were seeded in 6-well culturing plates and allowed to grow for 12 h. Then, they were treated with the test compound **8g** and allowed to incubate for 48 h. After the incubation period, all the steps were in accordance with the manufacturer's instruction, i.e., the removal of the medium, the washing of the cells with D-PBS twice, and the fixing of the cells with 2% paraformaldehyde. Then, the cells were permeabilized with 0.01% Tween 20 in D-PBS and 1% BSA, incubated with 50 μL of a caspase-3–FITC mixture (Abcam, ab65613) in dark at room temperature for another 60 min. Finally, the cells were washed, suspended in the PBS solution and analyzed for the expression of caspases by an EVOS FL AUTO fluorescence microscope.

Statistical analysis

Each sample was repeated thrice for the accuracy of its biological efficacy, and the average of all those values was considered as the final, where the data were shown as the mean ± SD. The statistical analysis was based on the Student's t-test, where the significant values ($p < 0.05$) are shown by * and the highly significant values ($p < 0.01$) are shown as ** against the untreated control measurements run using the Graphpad prism software (version 6).

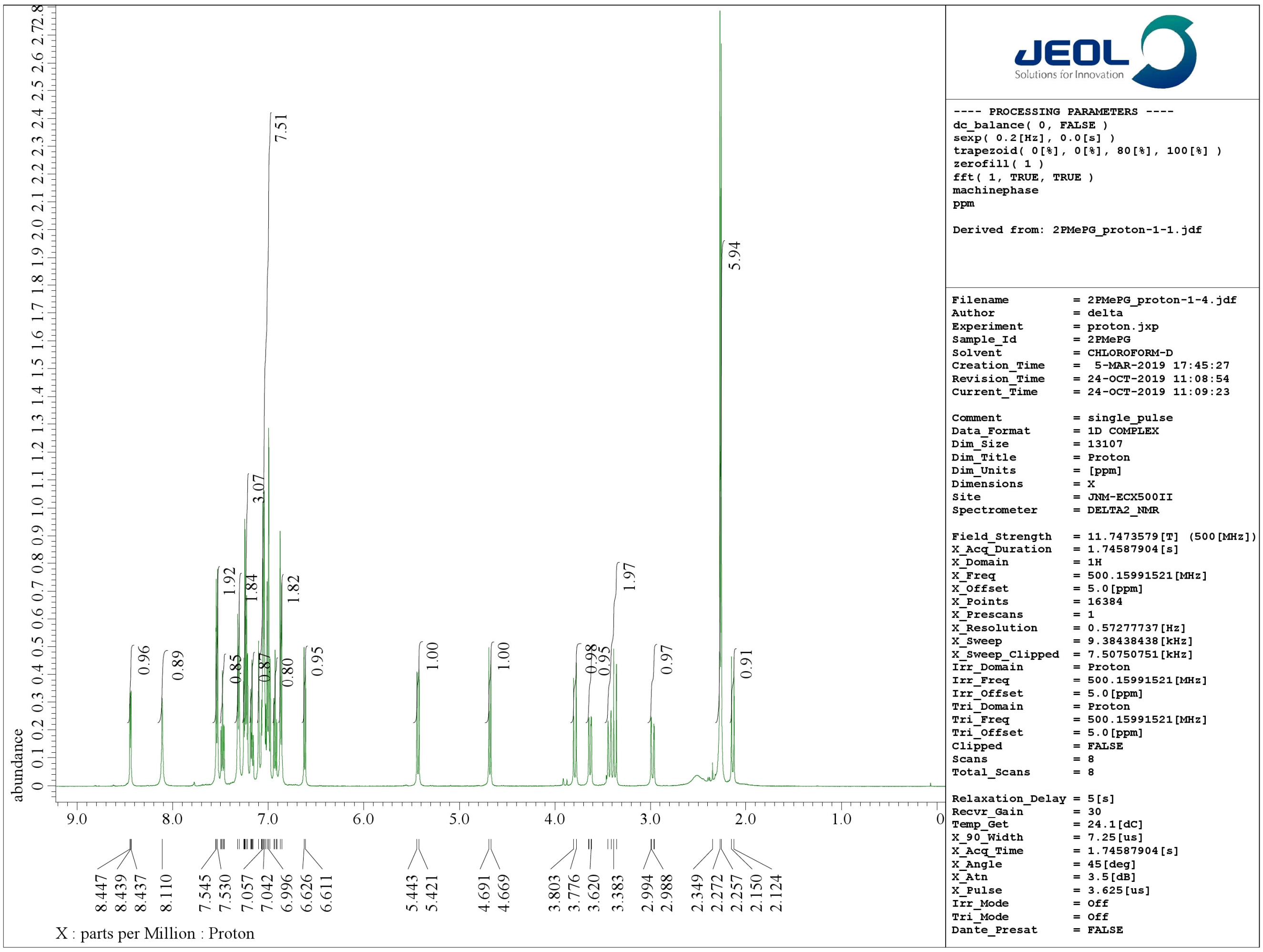

Figure 1. ^{1}H NMR spectrum of 8e.

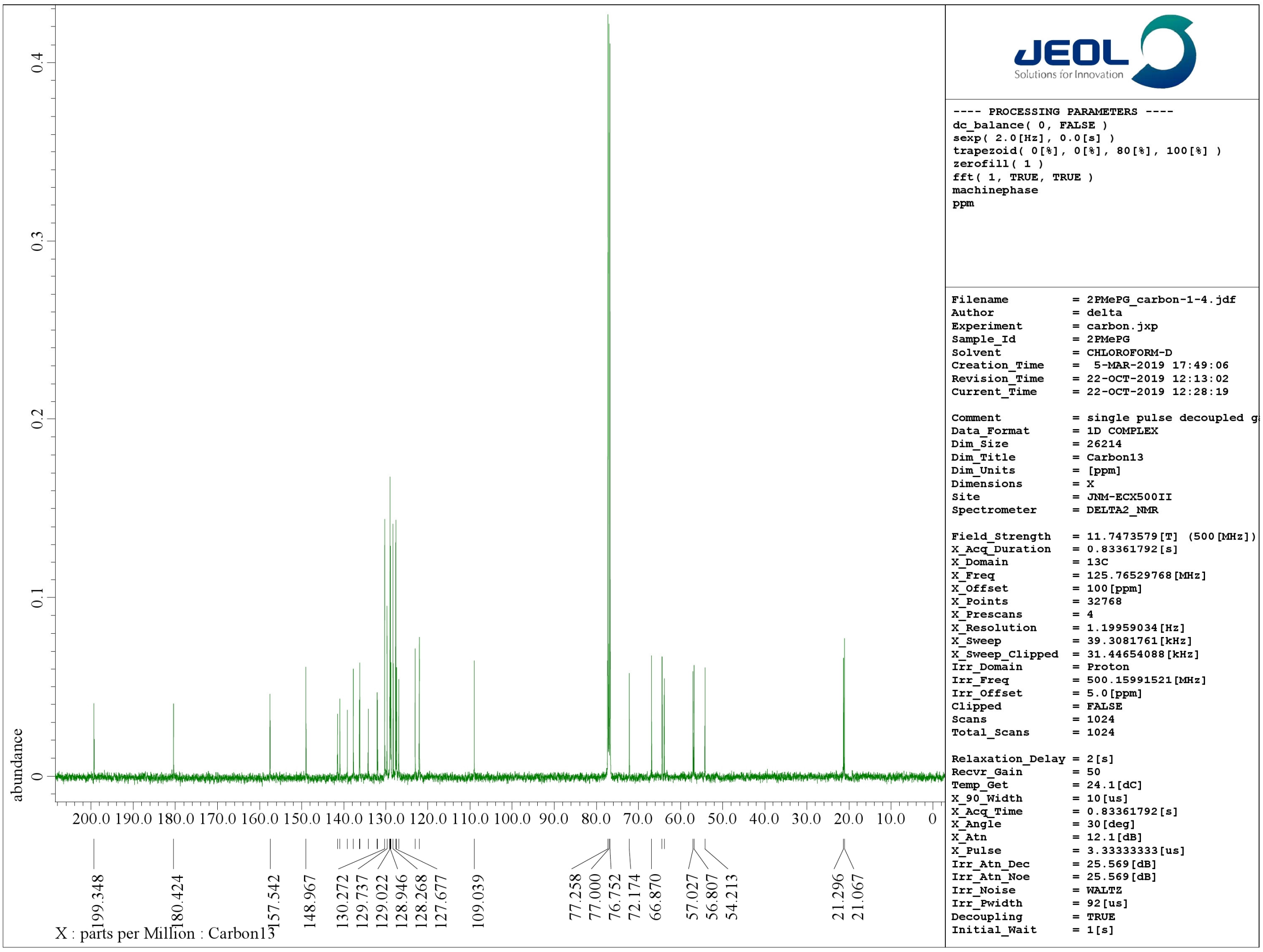

Figure 2. ^{13}C NMR spectrum of **8e**.

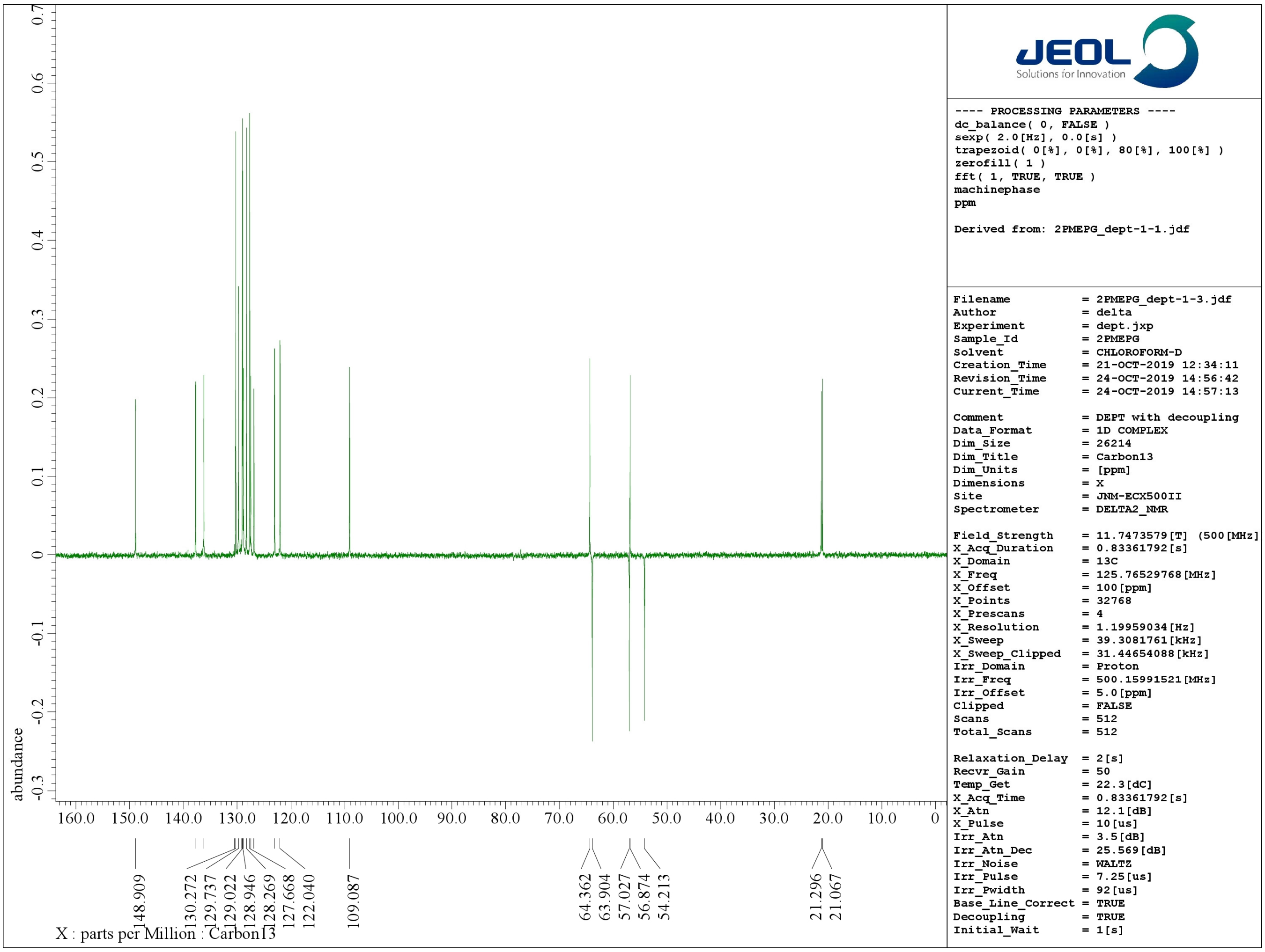

Figure 3. DEPT 135 NMR spectrum of **8e**.

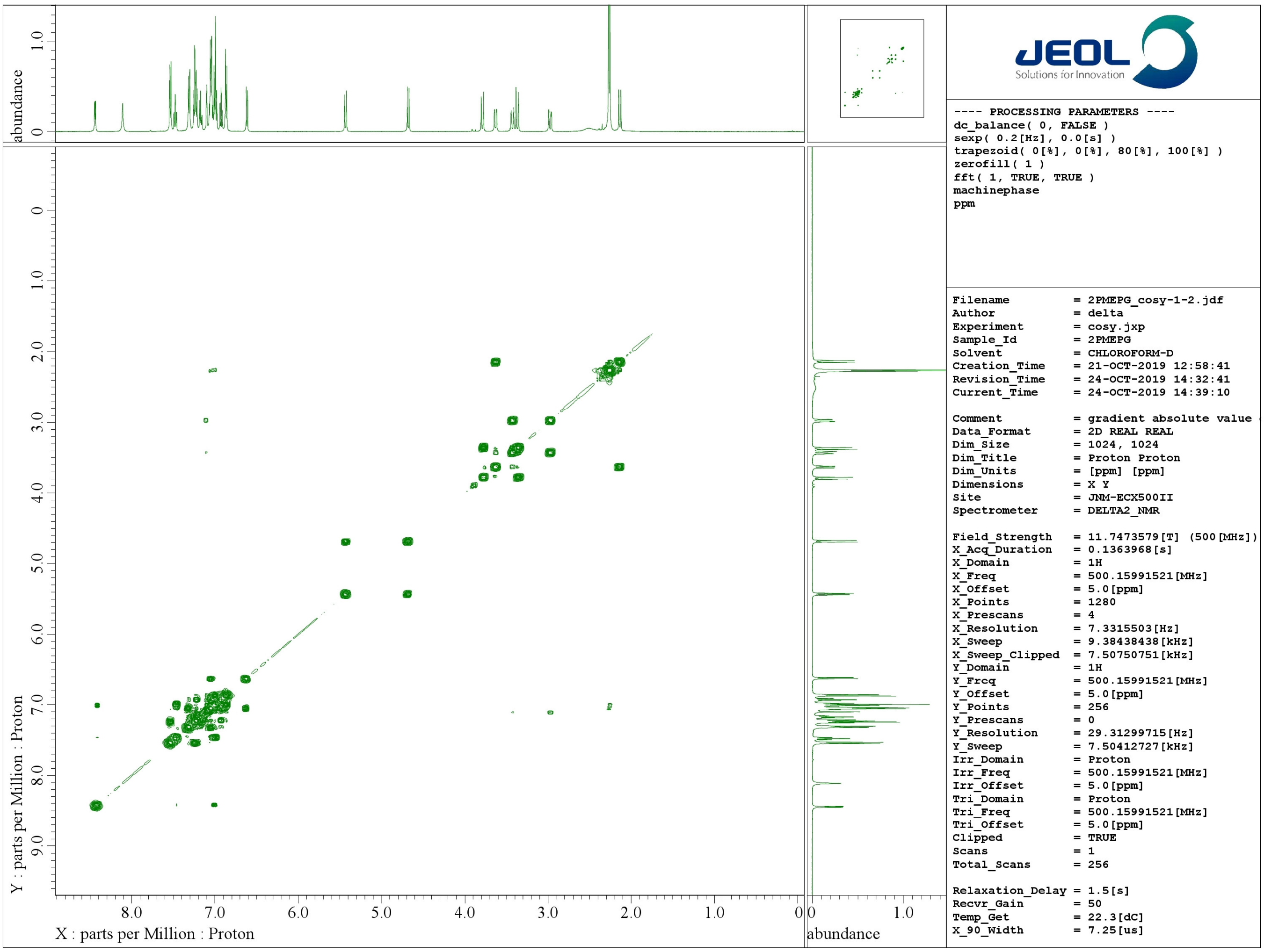

Figure 4. COSY NMR spectrum of **8e**.

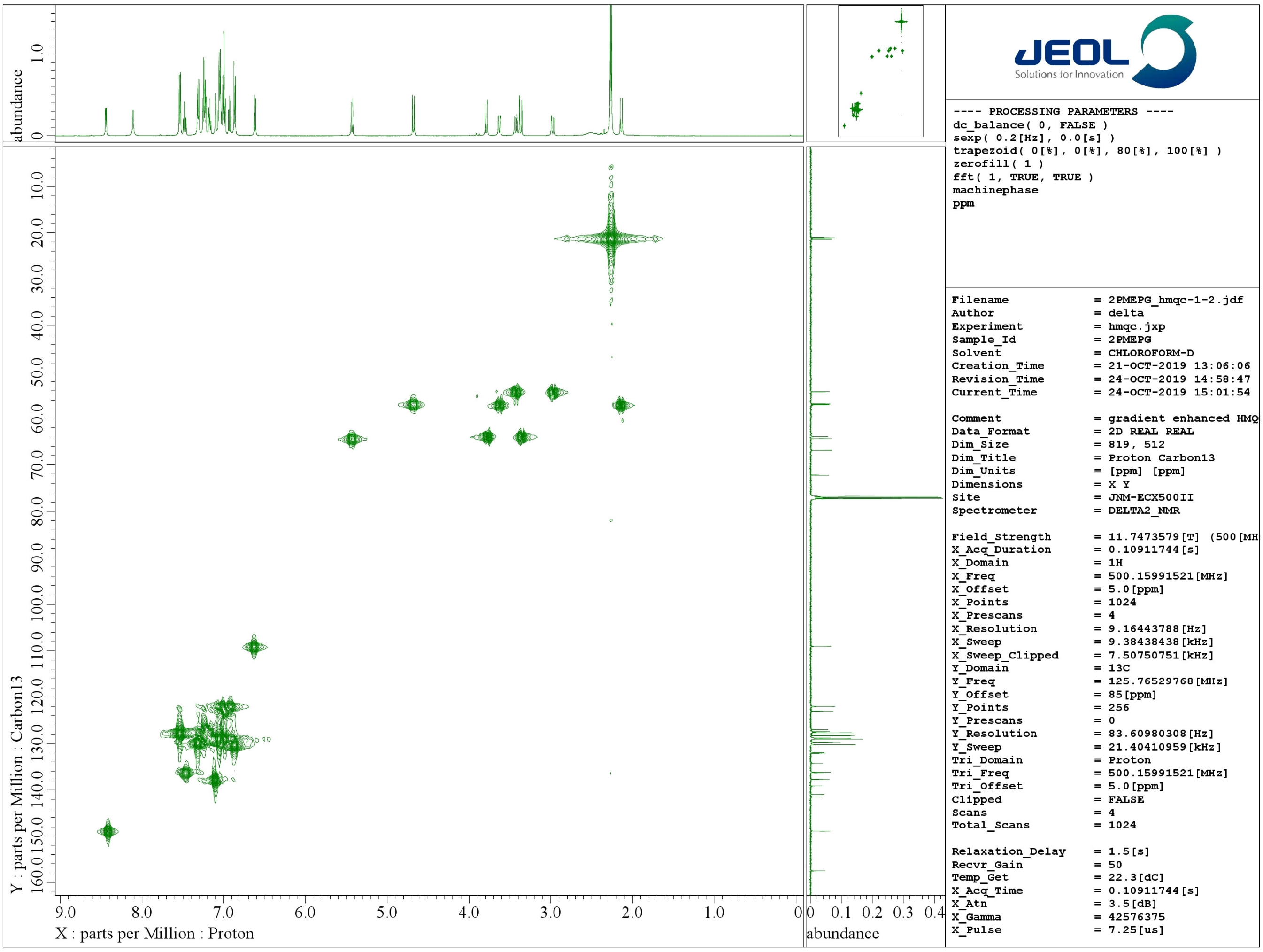

Figure 5. HMQC NMR spectrum of **8e**.

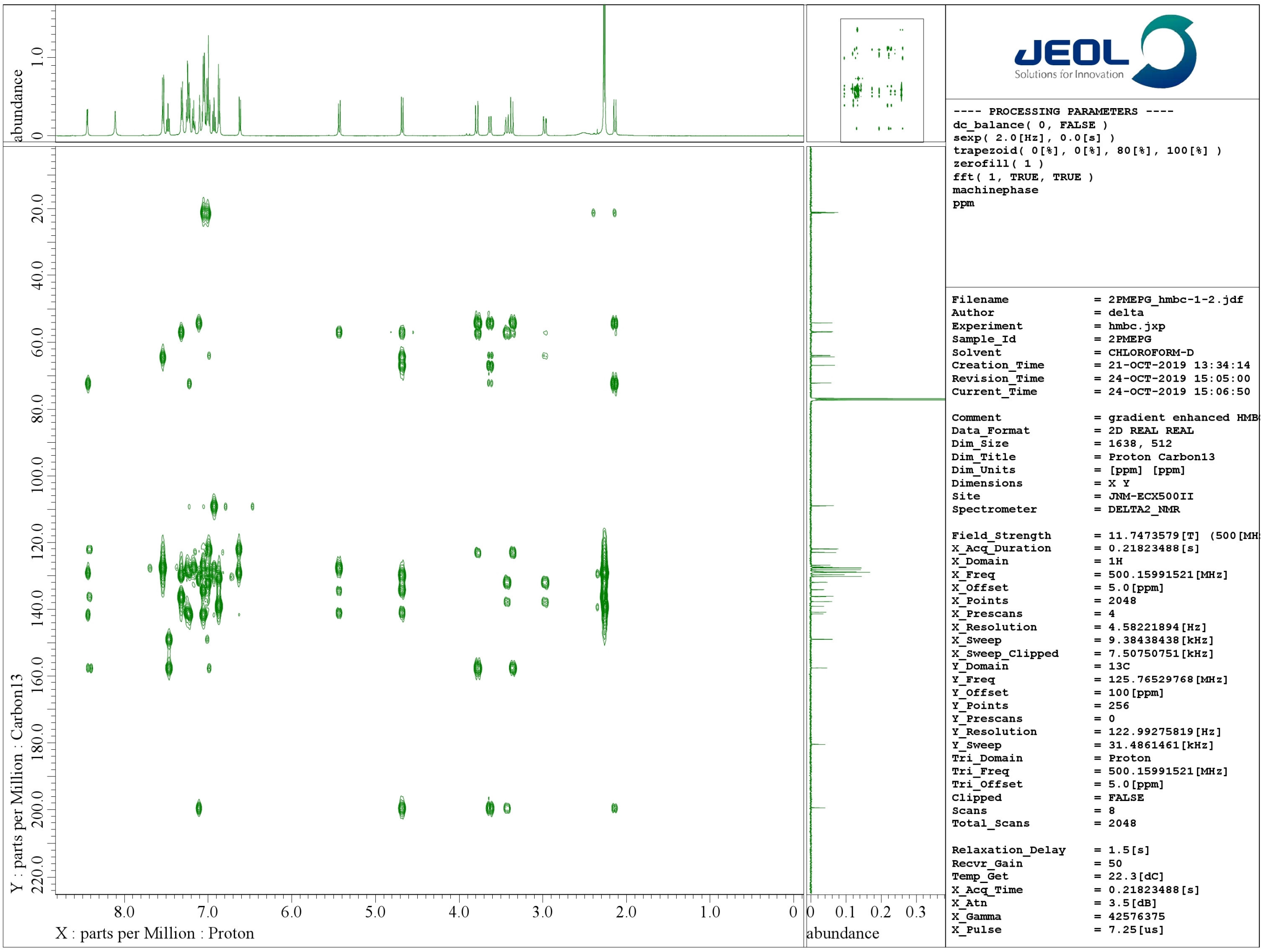

Figure 6. HMBC NMR spectrum of **8e**.

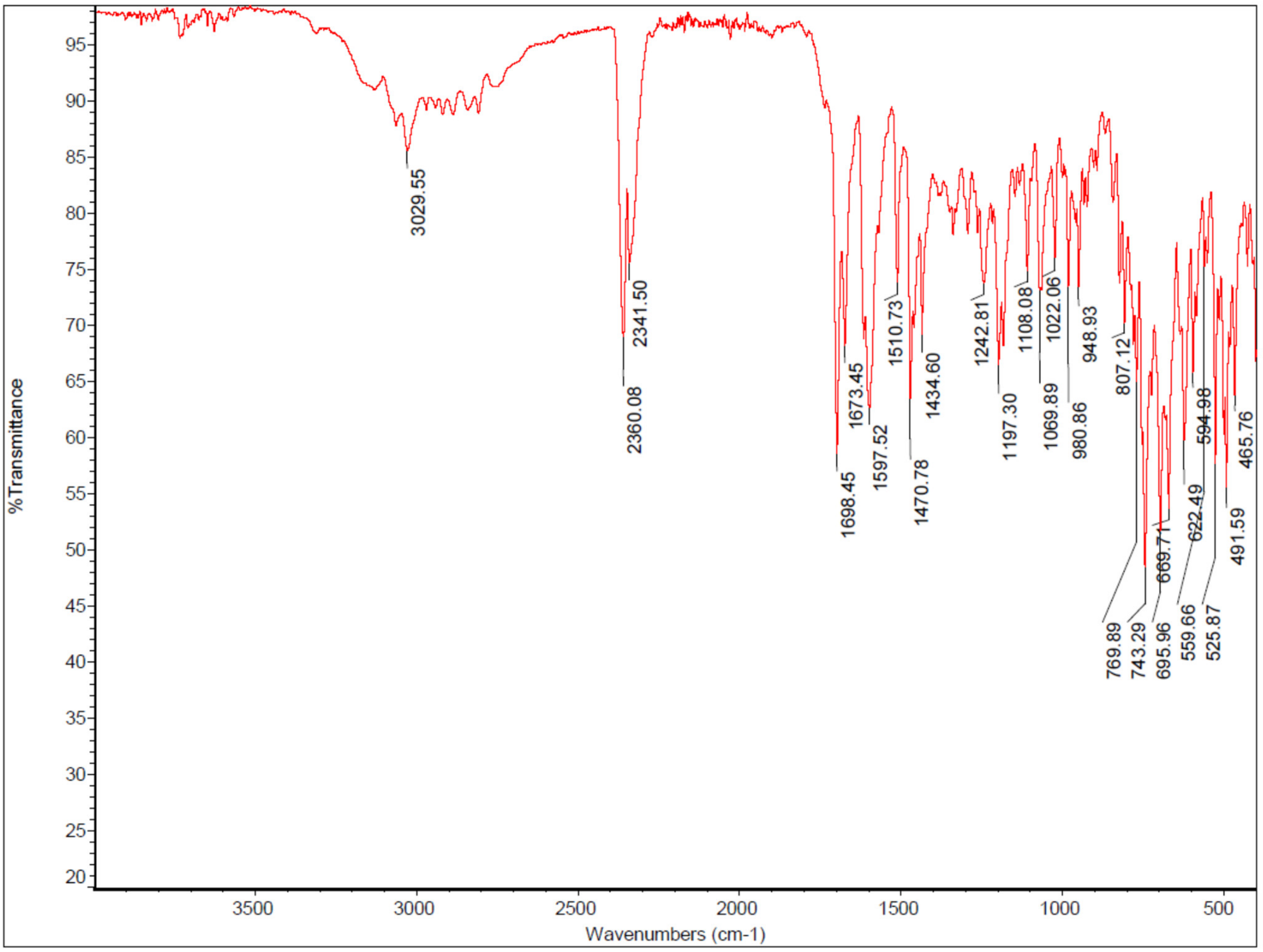

Figure 7. FT-IR spectrum of **8e**.

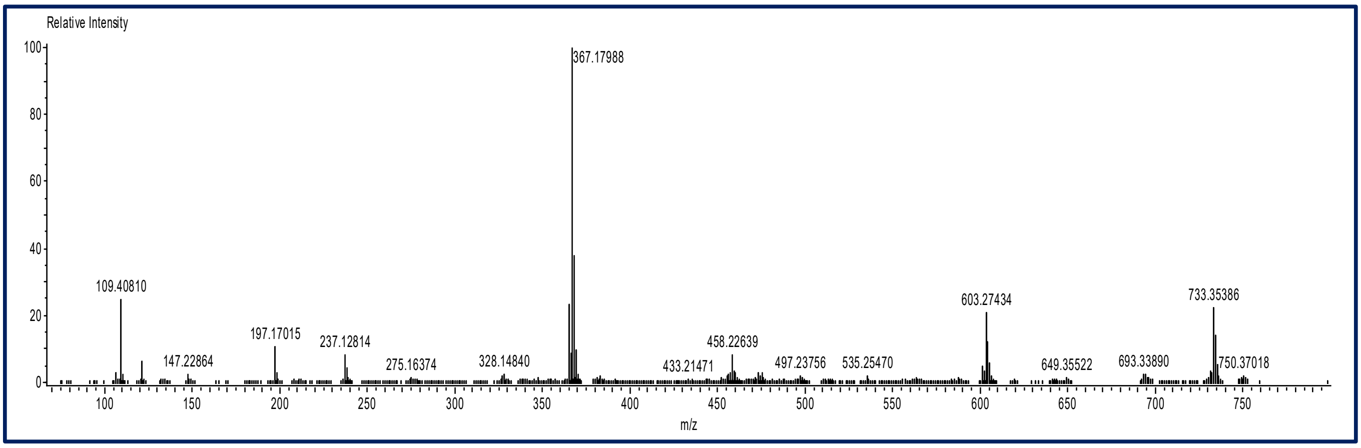

Figure 8. Mass spectrum of **8a**.
